# Pre-treatment minority HIV-1 drug resistance mutations and long term virological outcomes: is prediction possible?

**DOI:** 10.1186/s12985-016-0628-x

**Published:** 2016-10-12

**Authors:** M. L. Mzingwane, C. T. Tiemessen, K. L. Richter, S. H. Mayaphi, G. Hunt, S. M. Bowyer

**Affiliations:** 1 Department of Medical Virology, University of Pretoria, Pretoria, South Africa; 2 Department of Pathology, National University of Science & Technology, Faculty of Medicine, P. O Box AC939, Ascot, Bulawayo Zimbabwe; 3 Centre for HIV and Sexually Transmitted Infections, National Institute of communicable Diseases, Johannesburg, South Africa; 4 Faculty of Health Sciences, University of the Witwatersrand, Johannesburg, South Africa; 5 National Health Laboratory Services Tswane Academic Division, Pretoria, South Africa

**Keywords:** HIV, Virological outcomes, Drug resistance, Minority variants

## Abstract

**Background:**

Although the use of highly active antiretroviral therapy in HIV positive individuals has proved to be effective in suppressing the virus to below detection limits of commonly used assays, virological failure associated with drug resistance is still a major challenge in some settings. The prevalence and effect of pre-treatment resistance associated variants on virological outcomes may also be underestimated because of reliance on conventional population sequencing data which excludes minority species. We investigated long term virological outcomes and the prevalence and pattern of pre-treatment minority drug resistance mutations in individuals initiating HAART at a local HIV clinic.

**Methods:**

Patient’s records of viral load results and CD4 cell counts from routine treatment monitoring were used and additional pre-treatment blood samples for Sanger sequencing were obtained. A selection of pre-treatment samples from individuals who experienced virological failure were evaluated for minority resistance associated mutations to 1 % prevalence and compared to individuals who achieved viral suppression.

**Results:**

At least one viral load result after 6 months or more of treatment was available for 65 out of 78 individuals followed for up to 33 months. Twenty (30.8 %) of the 65 individuals had detectable viremia and eight (12.3 %) of them had virological failure (viral load > 1000 RNA copies/ml) after at least 6 months of HAART. Viral suppression, achieved by month 8 to month 13, was followed by low level viremia in 10.8 % of patients and virological failure in one patient after month 20. There was potentially reduced activity to Emtricitabine or Tenofovir in three out of the eight cases in which minority drug resistance associated variants were investigated but detectable viremia occurred in one of these cases while the activity of Efavirenz was generally reduced in all the eight cases.

**Conclusions:**

Early viral suppression was followed by low level viremia for some patients which may be an indication of failure to sustain viral suppression over time. The low level viremia may also be representing early stages of resistance development. The mutation patterns detected in the minority variants showed potential reduced drug sensitivity which highlights their potential to dominate after treatment initiation.

**Trial registration:**

Not applicable.

**Electronic supplementary material:**

The online version of this article (doi:10.1186/s12985-016-0628-x) contains supplementary material, which is available to authorized users.

## Background

Highly active antiretroviral therapy (HAART) has resulted in improved quality of life among people infected with human immunodeficiency virus (HIV) including reduced mortality and morbidity rates. However, virological failure caused by emergence of drug resistant variants still occurs in some individuals on HAART [1]. Some individuals experience virological failure after HAART initiation as they may harbour pre-treatment drug resistant viral species [2–4]. HIV treatment guidelines from developed countries recommend drug resistance testing before initiation of HAART [5, 6]. In developing countries pre-treatment screening for drug resistant species is however not done as part of treatment optimization due to limited resources. Pre-treatment drug resistance data are usually obtained through conventional population sequencing methods which do not detect low level viral species with a frequency of less than 20 % [7, 8] and this may underestimate prevalence figures and the effect that these variants have on treatment outcomes.

The effect of pre-treatment resistance associated minority variants on treatment outcomes is a subject of many studies [7–14]. While some studies have reported lack of strong association between drug resistance minority variants and treatment outcomes [9, 10], there is also strong evidence suggesting otherwise. Patients with no detectable drug resistance mutations using the Sanger method but with low level resistance mutations detected by sensitive methods were shown to have a more than double increased risk of virological failure after initiating HAART [11]. Minority drug resistant species have been shown to reduce the effectiveness of first line non-nucleoside reverse transcriptase inhibitor (NNTRI) based regimens which are the widely used regimens in developing countries [12].

Viral load (VL) testing in individuals on HAART is the method used to detect HIV replication and virological failure. The measure of a successful HAART program would be a sustained viral suppression over time in individuals on the program. The South Africa HIV management guidelines recommend VL testing 6 months after HAART initiation followed by VL testing at 12 months and every 12 months thereafter [15]. Baseline drug resistance testing is not recommended but pre-treatment drug resistance prevalence figures of less than 5 % have been reported in South Africa [16]. We investigated long term virological outcomes and the pattern of pre-treatment minority drug resistance mutations in individuals initiating HAART at a local HIV clinic.

## Methods

### Participants

Participants were recruited from Tshwane District Hospital HIV clinic in Pretoria central, South Africa between July 2013 and May 2014 after written informed consent and followed up for at least 12 months and up to 33 months. Eligible participants were HIV infected treatment naïve adults with CD4 cell counts < 350 cells/μl and/or World Health Organization (WHO) clinical stage 3 or stage 4. Participants were initiated on a NNRTI based regimen consisting of Efavirenz (EFV), Emtricitabine (FTC) and Tenofovir (TDF).

### Sample collection and RNA extraction

Samples were obtained before treatment initiation. Plasma was isolated from 10 to 15 ml of whole blood collected in EDTA tubes by centrifugation at 1600 g for 10 min and stored at −70 °C until required for RNA extraction. RNA was extracted from 210 μl of plasma using the QIAamp Viral RNA Mini kits (Qiagen) and eluted with 60 μl elution buffer.

### Viral load monitoring

Follow up HIV-1 VLs for treatment monitoring were done after at least 6 months of treatment using Abbott Real Time HIV-1 test (Abbott laboratories, Illinois, USA) with a detection limit of 40 RNA copies/ml. Suppression was defined as a VL < 50 RNA copies/ml. Virological failure was defined as a VL ≥ 1000 RNA copies/ml after at least 6 months of HAART.

### HIV genotypic resistance testing

A previously described method and primers was used for nucleic acid amplification and sequencing [17]. Superscript III reverse transcriptase enzyme (Life Technologies Corporation, California, USA) was used for cDNA synthesis and Platinum Taq enzyme (Life Technologies Corporation, California, USA) was used for the polymerase chain reaction (PCR). PCR primers targeted the protease (*PR*) gene and the first 300 codons of the reverse transcriptase (*RT*) gene (HXB2 nucleotide 2166–3440). BigDye Terminator V3.1 Cycle Sequencing Ready Reaction Kit (Applied Biosystems, Foster City, CA, USA) was used for sequencing reactions with two forward and two reverse primers and sequencing was done on the 3100 Automatic capillary sequencer (Applied Biosystems, Foster City, CA, USA). Sequences were edited and assembled on Sequencher V 4.5 (Gene Codes Corporation, USA). The Stanford HIVdb algorithm Version 7.0. (http://hivdb.stanford.edu/) was used for subtyping, resistance mutations interpretation and quality assessment.

### HIV deep sequencing

For ultra-deep sequencing (UDS) amplicons obtained from pre-treatment samples from four individuals who experienced virological failure were sent to Inqaba Biotechnical industries in Pretoria South Africa for sequencing up to 1 % prevalence. Additional samples from three individuals who virally suppressed and one sample from an individual who experienced low level viremia were also deep sequenced to provide comparison.

Briefly, cDNA samples were fragmented using an ultrasonication approach and the resulting fragments were purified and size selected, end repaired and an illumina specific adapter sequence ligated. Following quantification, the samples were individually indexed and a second size selection step was performed using AMPure XP Beads. Libraries were quality controlled on a DNA chip (Agilent 2100 Bioanalyzer) and then sequenced on illumina’s MiSeq platform, using a MiSeq v3 kit according to the manufacturer’s protocol. Fifty (50) Mb of data (2 × 300bp long paired end reads) were produced for each sample. This was followed by HIV sequence analysis and quality check, genotyping and drug resistance interpretations using Deepchek 1.4 HIV analysis software. A minimum of 461 sequences was required for 99 % confidence at 1 % threshold and a Q30 score measure was applied for basecalling.

### Pre-treatment drug resistance determination

Pre-treatment drug resistance was defined as identification of a mutation that is known to cause a reduced susceptibility to at least one prescribed drug.

### Adherence monitoring

Adherence was monitored using a combination of measures including checking for missed clinic visits, participant interviews and investigating reasons for which participants had been sent for counselling sessions.

### Statistical analysis

Differences between mean CD4 cell counts were calculated in excel using the *t*-test Two sample assuming equal variances.

## Results

### Enrolled participants characteristics and follow up

A total of 78 participants were recruited into the study. At least one follow up viral load result was available for 65 participants. Follow up viral loads were not available for 13 participants (16.6 %) due to loss to follow up. Participant characteristics are shown in Table 1.

**Table 1:** Enrolled participants characteristics

Participants (*n* = 78)	
Gender	
Males (%)	26 (33.3)
Females (%)	52 (66.7)
Age (years)	
Range	23–69
Mean age	35
Baseline CD4 counts (cells/μl)	
< 200	39
200–349	34
≥ 350	5
Mean CD4 count	192
Travel history/Stay outside South Africa	
None	56
Central Africa	2
North Africa	1
Southern Africa	28
West Africa	2
Europe	3
Years since HIV diagnosis	
< 1	61
1–5	15
> 5	2

### Treatment initiation to first viral load monitoring

**Fig. 1:**
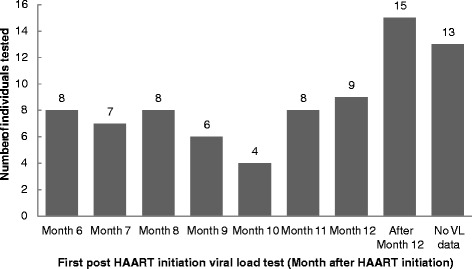
Time of first viral load testing after HAART initiation. The majority of participants (46) had at least one viral load (VL) test done by month 12 after HAART initiation. The first viral load test was done after month 12 in 15 (24.5 %) individuals. No viral load tests had been done by month 30 in 13 individuals

After treatment initiation, participants came for the first viral load monitoring at different time points (Fig. 1).

### Virological outcomes

**Fig. 2:**
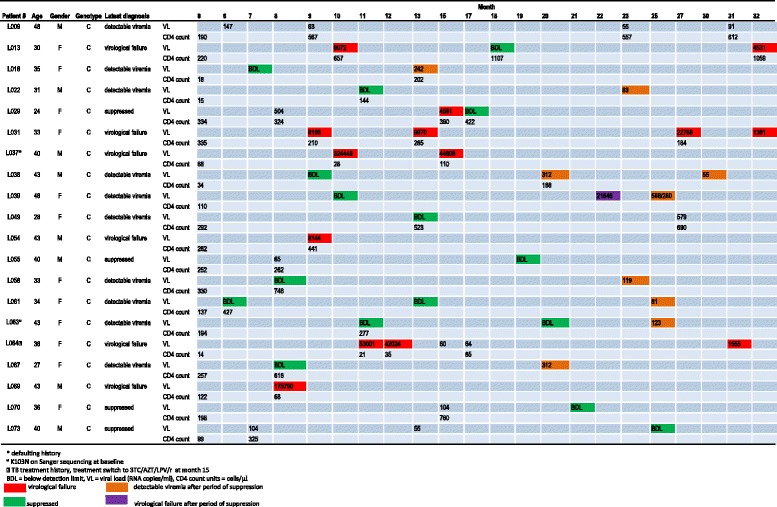
Participants with detectable viremia after at least 12 months of HAART. Virological failure generally occurred within the first 12 months of treatment initiation (*red squares*) while detectable viremia after initial suppression was shown to occur after month 20 of treatment (*orange squares*)

There were 20 participants (30.8 %) with detectable viremia at some point after at least 6 months of HAART (Fig. 2). Virological failure (>1000 RNA copies/ml) was detected in eight participants (12.3 %) with the following identification codes (IDs): L013, L029, L031, L037, L039, L054, L064 and L069 while the rest of the participants had low level viral loads < 1 000 RNA copies/ml. Virological failure occurred at 6–12 months after HAART initiation in six individuals (accounting for 9.2 % overall and 75 % in virological failure group) while virological failure occurred at 22 months in one individual (L039) who had initially achieved viral suppression.

There were eight participants (12.3 %), L022, L038, L039, L049, L058, L061, L063 and L067, who had initially suppressed the virus to below detection limits by month 8 to month 13 followed by detectable viremia at month 20 or after in all cases. The detected VLs after initial suppression were < 1 000 RNA copies/ml except for L039 with a VL of 21 646 RNA copies/ml. Subsequent viral load results after the initial rebound were available for two of these individuals, L038 and L039, and were done 10 and 3 months later respectively. The detectable viremia was shown to persist in both cases.

### Immunological outcomes of participants with detectable viremia

There were no differences in mean baseline CD4 cell counts between the group that managed to achieve viral suppression and the groups with detectable viremia (Table 2). The same pattern was observed in CD4 cell counts done after 6–12 months of treatment. However, there was a significant difference between mean baseline CD4 cell counts and mean CD4 cell counts at 6–12 months after treatment initiation for the virally suppressed group and the low level viremia group. The virological failure group did not achieve a significant change in mean CD4 cell counts between these two time points. CD4 cell counts at time of virological failure were < 500 cells/μl (range = 21–441 cells/ μl) for six out of the eight participants while participant L013 who was virally suppressed at some point had a CD4 cell count > 500 cells/μl (Fig. 2). Of the eight participants who initially suppressed the virus but later had detectable viremia, three had CD4 cell counts < 350 cells/μl at the time of viral suppression, while four participants had CD4 cell counts > 350 (range = 427–746 cells/μl) (Fig. 2).

**Table 2:** Comparison of mean CD4 cell counts: Intergroup and intragroup comparison of mean CD4 cell counts for the virally suppressed group, treatment failure group and low level viremia group at different time points

	Virally suppressed group	Virological failure group	Low level viremia group	Any viremia group	*P*-value (α = 5 %)
Baseline CD4 cell count (cells/μl)	*n* = 45, Mean = 199	*n* = 8, Mean = 183			0.74
	*n* = 45, Mean = 199		*n* = 12, Mean = 181		0.66
	*n* = 45, Mean = 199			*n* = 20, Mean = 182	0.61
		*n* = 8, Mean = 183	*n* = 12, Mean = 181		0.97
CD4 cell count @ 6–12 months after treatment initiation (cells/μl)	*n* = 18, Mean = 387	*n* = 8, Mean = 250			0.18
	*n* = 18, Mean = 387		*n* = 9, Mean = 438		0.58
	*n* = 18, Mean = 387			*n* = 17, Mean = 350	0.64
		*n* = 8, Mean = 250	*n* = 9, Mean = 438		0.07
*P*-value (α = 5 %)	0.0001	0.47	0.001	0.01	

### Baseline minority resistance mutations in participants with detectable viremia

**Fig. 3:**
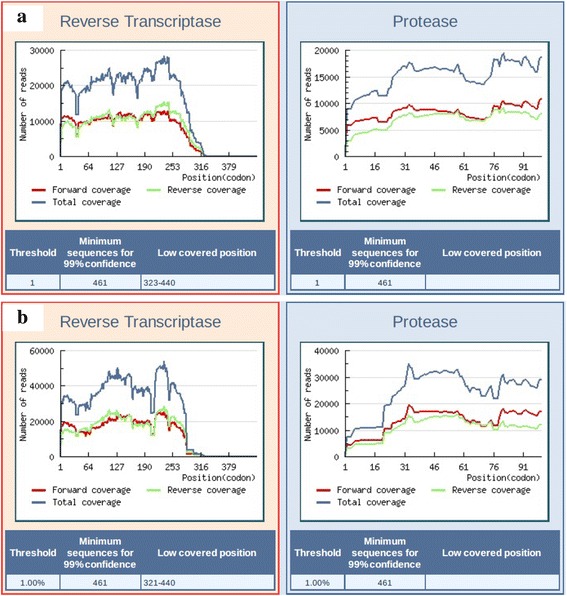
Deep sequencing coverage of analysed samples. **a** Showing L013 (virological failure) and (**b**) showing L001 (virally suppressed). Mutations were excluded from analysis for any of the following: noisy mutations filtering, coverage filtering, forward/reverse unbalanced frequency and forward/reverse unbalanced coverage. Additional file 1: Figure S1 shows the rest of the samples. C – E (virological failure, L031, L054 and L064 respectively) and F – H (detectable viremia, L009 and virally suppressed L074 and L075 respectively)

Detection of baseline minority resistance associated mutations using ultra deep sequencing to 1 % threshold was done for eight participants. The coverage and basis for excluding some mutations from analysis is shown in Fig. 3 and Additional file 1: Figure S1. Of these eight participants: four had virological failure (>1000 RNA copies/ml), one had low level viremia < 1 000 RNA copies/ml and three had suppressed viremia (<50 RNA copies/ml) after at least 6 months of HAART. The mutations list and patterns are shown in Table 3.

**Table 3:** Baseline minority resistance mutations detected by ultra-deep sequencing

ID	Virological outcome after at least 6 months of HAART	Genotype	Baseline resistance mutations (% prevalence)			Drug activity of initiated regimen (Based on drug resistance mutation surveillance list [31])	Drug activity of initiated regimen (Based on impact of treatment efficacy Stanford HIVdb algorithm)
			NRTIs	NNRTIs	TAMS		
L001	Suppressed	C	D67E (1.67) T69N (1.25) F77L (2.16)	P225H (1.35) F227L (2.36)	1	TDF and FTC resistance EFV resistance	TDF susceptible FTC susceptible EFV resistance
L009	Detectable viremia (<1000 RNA copies/ml)	C	D67E (1.29) T69N (1.03) F77L (1.15) L100I (1.15) KI03N (4.17)	F227L (1.49)	1	TDF and FTC resistance EFV resistance	TDF susceptible FTC susceptible EFV high level resistance
L013	Virological failure	C	D67E (1.73) T69N (1.21) F77L (1.13)	P225H (1.16) F227L (1.98)	1	TDF and FTC resistance EFV resistance	TDF susceptible FTC susceptible EFV low level resistance
L031	Virological failure	C	D67E (1.05) F77L (1.02)	F227L (1.78)	1	TDF and FTC susceptible EFV resistance	TDF susceptible FTC susceptible EFV resistance
L054	Virological failure	C	A62V (1.51) D67E (1.16) F77L (1.52)	F227L (1.26)	1	TDF resistance and FTC susceptible EFV resistance	TDF resistance FTC susceptible EFV resistance
L064	Virological failure	C	D67E (1.32)	F227L (1.43)	1	TDF and FTC susceptible EFV susceptible	TDF susceptible FTC susceptible EFV resistance
L074	Suppressed	CRF37_cpx	D67E (1.72) T69N (1.34) M184I (1.04)	P225H (1.12) F227L (1.86)	1	TDF and FTC resistance EFV resistance	TDF susceptible FTC resistance EFV resistance
L075	Suppressed	CRF37_cpx	D67E (1.83) T69N (1.58) L74V (1.23) F77L (1.01) M184I (1.44)	P225H (2.22) F227L (1.47)	1	TDF and FTC resistance EFV resistance	TDF susceptible FTC resistance EFV low level resistance

### Impact of treatment efficacy of initiated drugs

NRTI drugs TDF or FTC had potentially reduced activity in three out of the eight cases in which minority drug resistance associated variants were detected but detectable viremia occurred in one of these cases. The activity of EFV was reduced in all the eight cases ranging from low level resistance (two cases) to high level resistance (1 case). Taken together, the pattern of minority variants detected showed potentially reduced EFV treatment efficacy and were generally susceptible to the NRTI drugs TDF and FTC at 1 % prevalence.

## Discussion

The goal of HIV antiretroviral therapy is to achieve sustained viral suppression in individuals on treatment. Virological failure due to development of drug resistance is still a challenge for many HIV treatment programmes in developing countries due to lack of resources for effective treatment monitoring and treatment optimization among other challenges. Furthermore, the effect of pre-treatment resistance associated minority variants on virological outcomes is still not well understood and largely underestimated because of reliance on conventional population sequencing data which do not include minority species. In this long term follow up study, baseline samples showed a high prevalence of pre-treatment resistance associated minority variants although their clinical relevance requires further study. There was high prevalence (30.8 %) of detectable viremia of any kind after at least 6 months of HAART and in 40 % of these cases detectable viremia occurred after previous viral suppression to below detection limits.

Virological failure occurred in 12.3 % of the participants compared to an overall 15 % virological failure rate (range 0–43 %) calculated from 19 studies done in sub-Saharan Africa countries [18]. However the duration of treatment at time of failure detection was not mentioned for most of these studies. We detected virological failure at month 6 to 12 after initiation of HAART except where virological failure was detected after previous viral suppression. Viral load testing at month 6 and month 12 after HAART initiation, which is recommended in South African public health institutions caring for HIV positive patients, seems adequate to detect early virological failure as was shown in our participants. However, up to 23.1 % of participants as indicated in Fig. 1 had their first post HAART initiation viral load after more than 12 months which may affect the detection of early virological failure. South Africa HIV management guidelines [15] use a cut off value of VL > 1000 RNA copies/ml to define virological failure, however HIV treatment guidelines from some developed countries are more stringent and define virological failure as confirmed VL > 50 RNA copies/ ml [5, 6]. Using this criterion, our frequency of virological failure would increase by more than 50 % from 12.3 to 30.8 %.

We noted that viral suppression had been achieved by month 8 to month 13 and viral rebound occurred after month 20 in a subset of individuals indicating failure to sustain viral suppression over time. Although the rebound resulted in low level viremia ranging from 83 RNA copies/ml to 579 RNA copies/ml in seven of the cases, detection of low level viremia after previous viral suppression should be monitored as these detections may be early stages of resistance development.

The association of pre-treatment minority drug resistance associated HIV variants of < 1 % frequency or greater with increased risk of virological failure for individuals on NNRTI based regimens has been highlighted [12]. The mutation patterns detected in the minority variants showed potentially reduced sensitivity to EFV and to a lesser extent TDF and FTC which highlights their potential to dominate after treatment initiation. However, the comparison of the pre-treatment pattern of minority variants detected between the virological failure group and the suppressed group did not show a specific pattern associated with virological failure at this stage indicating that other factors might be involved. Factors such as mutation linkages and mutational loads also need to be investigated. A single TAM D67E was found to be highly prevalent but TDF-based regimens have been shown to be effective in the presence of other single TAMs such as M41L at baseline [19]. Pre-treatment minority TAMs M41L and K70R have been reported in other studies [20] including multiple TAMs of up to six in another study [13].

Pre-treatment HIV drug resistance has been shown to increase regimen switches in developing countries [2] which results in increased treatment costs and exhaustion of treatment options. Recent studies have also demonstrated strong association between low level viremia of 200–499 RNA copies/ml with virological failure [21] while some studies have associated virological failure with lower levels of less than 50 copies per ml [22] after a follow up period of up to 2 years. This compares with our results where viremia after initial suppression was detected at month 20 and after and shown to persist on subsequent testing. Low level viremia may also be viewed in light of the correlation between viral persistence and the viral reservoir size which may require several years of HAART to reduce [23, 24].

We noted that 43 % of individuals who experienced low level viral rebound after initially suppressing the virus generally had poor immunological outcomes with a CD4 cell count < 350 cells/μl after more than 6 months of HAART. The same poor immunological outcome pattern was observed in individuals who experienced virological failure where 75 % of them had a CD4 cell count < 350 cells/μl. However CD4 cell count monitoring in isolation has been shown to be a poor marker for treatment failure [25, 26] and in our case we had a minority of individuals with CD4 cell counts > 500 experiencing virological failure or viral rebound.

The study has a number of limitations in particular the absence of resistance data at the time of virological failure. Additional samples taken at the time of routine viral load testing were not always available since the researchers were not part of the routine patient care personnel at the clinic where participants accessed care. The small number of samples that were deep sequenced also limited the information and conclusions that could be derived from this data. The data obtained however provide a basis for further investigation using larger sample sizes and comparison of baseline minority mutations with sequences dominating at time of failure. We also relied on self-reported information on treatment adherence to monitor participant’s treatment adherence levels. Poor adherence to treatment may have been the cause of virological failure in some individuals given that there was a high loss to follow up of 20 % in our cohort. Many studies have shown that poor treatment adherence is associated with virological failure in resource limited settings [27–30] and that an adherence of at least 95 % in individuals with minority variants significantly lowers the risk of virological failure [12].

## Conclusions

While the development of low level viremia or virological failure after previously suppressing the virus that we noticed in a subgroup of our participants could be due to viral persistence emanating from established viral reservoirs, there is need to establish if such occurrences might be due to resistant minority variants beginning to take over and dominate following suppression of the treatment sensitive population. These data therefore highlight the need for a better understanding of the role played by pre-treatment HIV drug resistance associated minority variants under various clinical settings.

## Additional file

Additional file 1: Figure S1. Deep sequencing coverage. C – E shows sequencing coverage for samples with virologicalfailure (L031, L054 and L064 respectively), F shows coverage for a sample with detectable viremia (L009)and G and H show coverage for virally suppressed samples (L074 and L075 respectively). Mutations wereexcluded from analysis for any of the following: noisy mutations filtering, coverage filtering, forward/reverse unbalanced frequency and forward/reverse unbalanced coverage. (DOCX 1189 kb)
