# Supplementary figures and images for: Pre-treatment minority HIV-1 drug resistance mutations and long term virological outcomes: is prediction possible?
Source: Virol J. 2016 Oct 12;13:170. doi: 10.1186/s12985-016-0628-x (PMC5062819; doi:10.1186/s12985-016-0628-x)

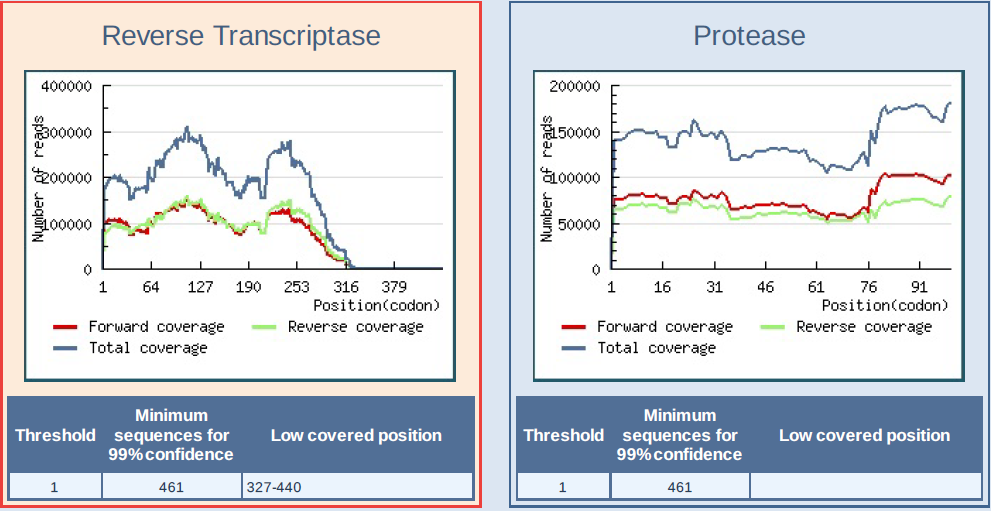


**(C)**


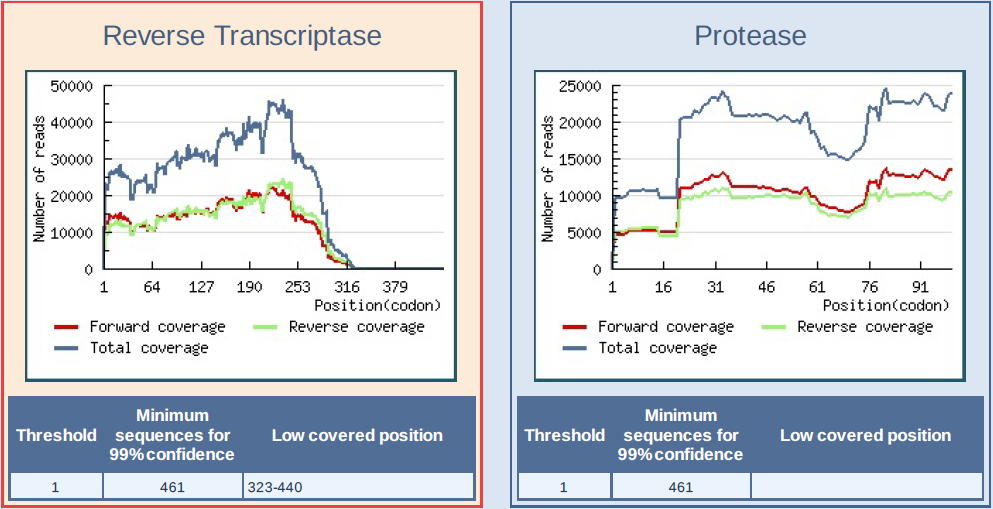


**(D)**


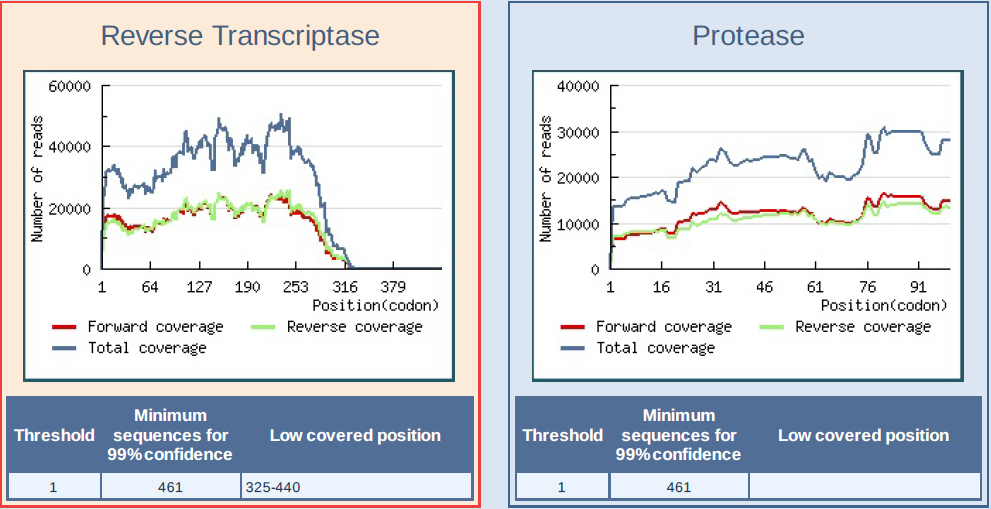


**(E)**


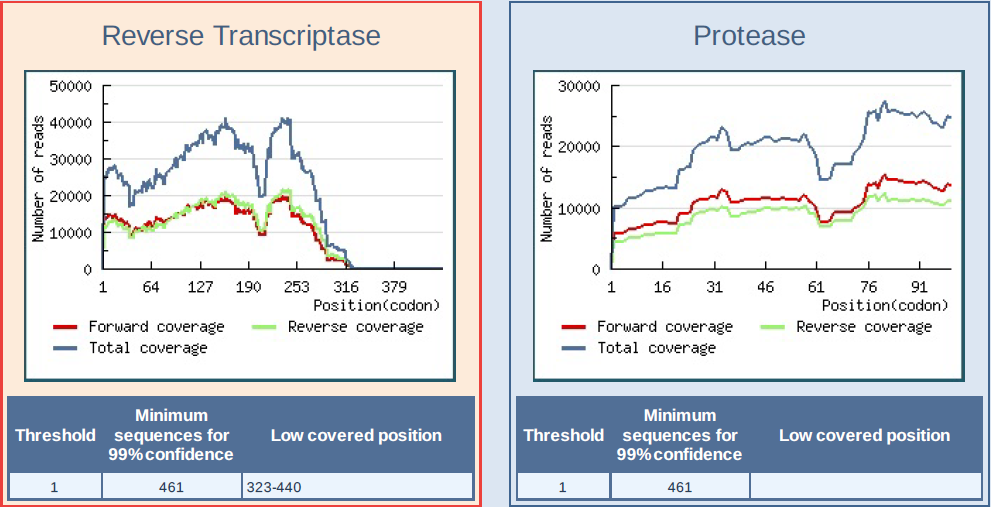


**(F)**


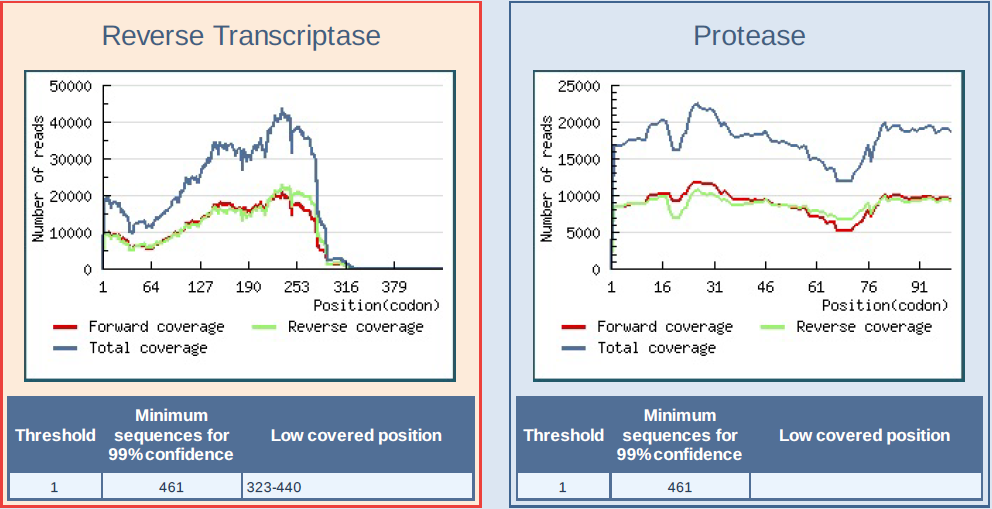


**(G)**


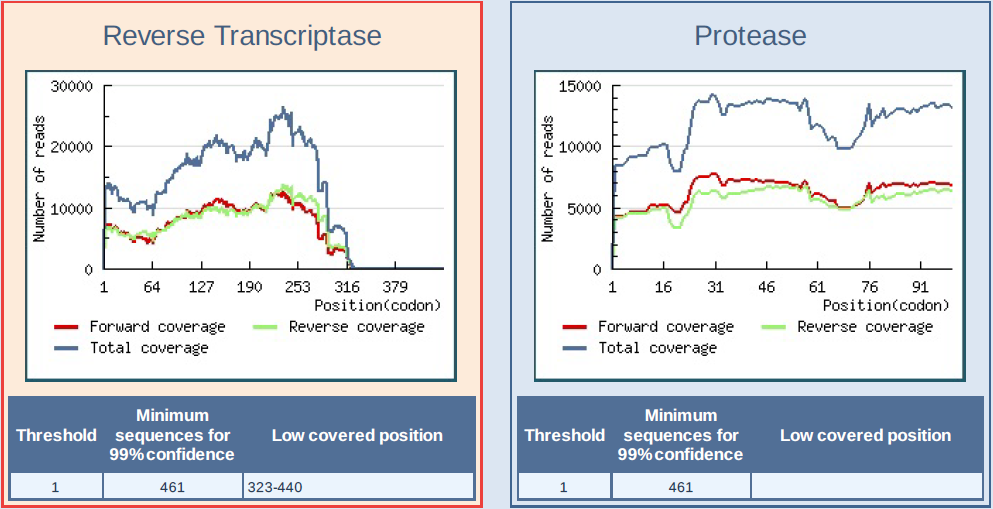


**(H)**

Supplement: Additional file 1: Figure S1. — Deep sequencing coverage. C – E shows sequencing coverage for samples with virologicalfailure (L031, L054 and L064 respectively), F shows coverage for a sample with detectable viremia (L009)and G and H show coverage for virally suppressed samples (L074 and L075 respectively). Mutations wereexcluded from analysis for any of the following: noisy mutations filtering, coverage filtering, forward/reverse unbalanced frequency and forward/reverse unbalanced coverage. (DOCX 1189 kb) [file 12985_2016_628_MOESM1_ESM.docx]
